# Effect of Chinese Herbs on Serum Biochemical Parameters, Immunity Indices, Antioxidant Capacity and Metabolomics in Early Weaned Yak Calves

**DOI:** 10.3390/ani12172228

**Published:** 2022-08-29

**Authors:** Cuixia Jiang, Quanmin Dong, Xiaoping Xin, Abraham Allan Degen, Luming Ding

**Affiliations:** 1 State Key Laboratory of Grassland Agro-ecosystem, College of Ecology, Lanzhou University, Lanzhou 730000, China; 2 Qinghai Provincial Key Laboratory of Adaptive Management on Alpine Grassland, Qinghai University, Xining 810016, China; 3 Institute of Agricultural Resources and Regional Planning, Chinese Academy of Agricultural Sciences, Beijing 100081, China; 4 Desert Animal Adaptations and Husbandry, Wyler Department of Dryland Agriculture, Blaustein Institutes for Desert Research, Ben-Gurion University of the Negev, Beer Sheva 84105, Israel; 5 Institute of Qinghai-Tibetan Plateau, Southwest Minzu University, Chengdu 610041, China

**Keywords:** herbal extract, functional food, grazing, healthy feeding, sustainability, secondary metabolites

## Abstract

**Simple Summary:**

Herbs, as supplementary feed, have shown positive effects on livestock. The yak is a unique animal on the Qinghai-Tibetan plateau, and plays important roles in local livelihoods and ecology. Because of the harsh climate conditions, early weaned yak calves often face environmental and nutritional stress. The herbal active ingredients of some herbs improve the antioxidant capacity and immunity of animals. In the current study, early-weaned yak calves were supplemented with three widely used Chinese traditional herbal root extracts to examine whether they can improve the immune response and antioxidant capacity. The results demonstrated that the supplementary herbs increased the serum antioxidant capacity, and improved the energy and nitrogen metabolism of the yak calves.

**Abstract:**

Chinese traditional herbs are used widely as feed supplements to improve the immune response and antioxidant capacity of livestock. Twenty early-weaned 4-month-old yak calves (72.3 ± 3.65 kg) were divided randomly into four groups (n = 5 per group); three groups were provided with supplementary 80 mL/kg DMI of the root water extracts of either *Angelica sinensis*, *Codonopsis pilosula* or *Glycyrrhiza uralensis*, and one group (control) was not provided with a supplement. Compared to control calves, calves consuming the three herbal extracts increased serum concentrations of albumin (ALB) and glutathione peroxidase (GSH-Px), but decreased serum concentrations of free fatty acids (FFAs) and malondialdehyde (MDA) (*p* < 0.05). Calves consuming *A. sinensis* decreased (*p* < 0.05) serum concentration of total cholesterol (TC), and increased (*p* < 0.05) serum concentration of total proteins (TP). Serum FFA concentrations increased (*p* = 0.004) linearly with time in the control group, but not in the groups consuming herbs. Serum metabolomic data demonstrated that *A. sinensis* and *C. pilosula* regulate mainly amino acid metabolism, while *G. uralensis* regulates mainly carbon and amino acid metabolism. It was concluded that the three herbal root extracts, as dietary supplements, improved energy and nitrogen metabolism, and enhanced the antioxidant capacity of yak calves.

## 1. Introduction

With the increasing global demand for safe and healthy animal products, natural botanical feed additives are being sought to reduce the heavy use of chemicals and antibiotics, many of which are banned in some countries. Some traditional widely used Chinese herbs could potentially replace some of these banned substances. Chinese herbs have a long folk history as feed additives to cure illnesses, promote growth, and improve immunity. They also serve as antioxidant and anti-pathogen agents [1]. *Angelica sinensis*, *Codonopsis pilosula*, and *Glycyrrhiza uralensis* are three widely used native herbs growing in harsh alpine and arid environments in northwest China. Their roots are used for medical purposes. *Angelica sinensis* contains a variety of active ingredients, including polysaccharides, volatile oils, organic acids, and phthalides, which are reputed to protect the heart, enhance immune responses, and prevent myocardial infarction, arrhythmia, and atherosclerosis [2]. Ligustilide is the main active ingredient of volatile oils, and has antioxidant properties by suppressing reactive oxygen species (ROS) generation and lipid oxidation [3,4,5]. *Codonopsis pilosula* contains alkaloids, alkynes, terpenes, flavonoids, and saccharides, which protect the gastrointestinal tract, promote circulation, remove stasis, enhance immunity responses, and delay senescence [6]. *Glycyrrhiza uralensis* is traditionally used as a harmonizing agent with antioxidant, anti-inflammatory, chemo-preventive, and anti-proliferative activities [7]. More than 400 compounds have been isolated from *G. uralensis*, including triterpenoid saponins and phenolic compounds such as flavonoids, chalcones, and coumarins [8]. Adrenocorticomimetic effects were demonstrated by the major component, glycyrrhizin [9]. 

Beneficial effects have been reported for ruminants consuming herbal plants or mixtures of the plants. For example, feed digestibility and nutrient utilization were improved in ruminants consuming herbs [10,11]. Few studies, however, have examined the effect of water extract on the roots of *A. sinensis*, *C. pilosula*, and *G. uralensis* as feed additives for livestock. Water extracts contain most of the water-soluble ingredients, such as saccharides, flavonoids, and saponins, which are traditionally administered to humans. The early weaning of yak calves was introduced to improve the growth rate of calves and the reproductive performance of dams [12,13]. However, because of the harsh environment, namely, low air temperature, hypoxia, and strong ultra-violet light, and low nutrition, early-weaned yak calves are susceptible to diseases, which could lead to low growth rate or even death [13]. In early-weaned yak calves, there was an initial increase in serum white blood cells, indicating an immune response [14]. 

In a companion paper on early-weaned yak calves receiving *A. sinensis*, *C. pilosula*, and *G. uralensis* as feed additives, dry matter intake (DMI) in calves consuming the three herbs did not differ from control calves and ranged between 2.12 and 2.26 kg/d. Average daily gain increased in yak calves consuming *C. pilosula* root extract, while the ruminal molar proportions of propionate and isovalerate increased and rumen microbiota were altered in yak calves consuming the three herbal root extracts [15]. The current study is the first to examine the effects of the three herbal water extracts concomitantly on serum biochemical parameters, immunity indices, antioxidant capacity, and metabolomics in early-weaned yak calves. 

## 2. Materials and Methods

The protocol and all procedures on the yak calves were approved by the Animal Welfare and Ethics Committee of Lanzhou University (No. LZU20191024). 

### 2.1. Experimental Site 

**Figure 1:**
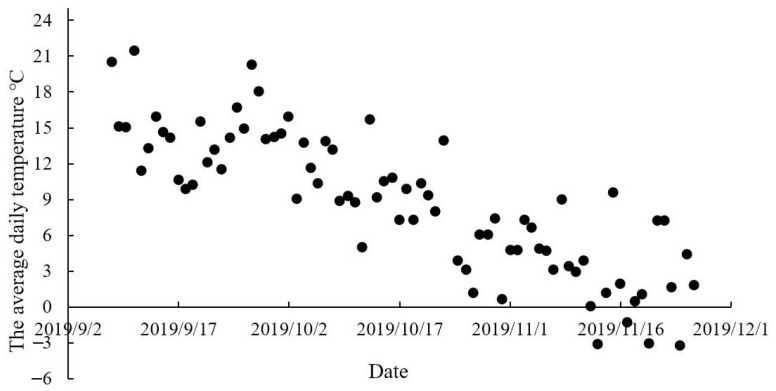
The average daily air temperature throughout the experimental period.

The study was carried out in Qinghai Province (36°55′ N,108°57′ E; 3170 m above sea level) between September and November, 2019. The daily air temperature ranged between −13.9 °C and 27 °C (Figure 1) during the study.

### 2.2. Preparation of Herbal Water Extracts

Sun-dried roots of two-year-old *A. sinensis*, *C. pilosula*, and *G. uralensis* were purchased in 2018 (Medicinal Trading Market, Longxi County, Gansu Province), and washed, dried, and ground to 1 mm in a hammer crusher (DF-50-A, Linda Machinery Co., LTD, Wenling, China). The leaching solution for roots of the herbs has been described earlier [16]. The preparation of the herbal extracts from the dry root powder followed Jiang et al. (2021) [15]. Thirteen batches of each herbal extract were prepared to span the needs of the entire study.

### 2.3. Experimental Design

Twenty, four-month-old weaned yak calves (72.3 ± 3.65 kg), 8 males and 12 females, were kept in one pen for one week with free access to concentrate, oat hay (2–3 cm length), and fresh water. Then, the calves were penned (2 m × 4 m) individually, divided into four groups (n = 5 for each group), matched for sex, and offered either no supplement (control group) or 80 mL/kg DMI of herbal root extract of either *A. sinensis*, *C. pilosula*, or *G. uralensis*. The root extract consumed by the calves was 3.12 g/kg DMI for *A. sinensis*, 3.19 g/kg DMI for *C. pilosula*, and 2.14 g/kg DMI for *G. uralensis*. 

The study included a 15-day acclimation period for the yak calves and a 60-day measurement period. Each calf consumed approximately 170 mL of herbal root water extract per day, which was mixed with 300 g of concentrate. The actual daily dosage of root water extract was based on the average daily DMI in the previous two weeks, and was consumed completely by the calves at 07:00. Then the calves had free access to oat hay and concentrate, which were provided separately, and at 18:00, concentrate, oat hay, and fresh water were added. 

### 2.4. Sampling Procedure

Herbal extracts were collected after each preparation and stored at −20 °C. Ten milliliters of jugular vein blood were collected in vacuum tubes on d 15, 30, 45, and 60 before morning feeding. The blood was centrifuged at 3000× *g* at 4 °C for 15 min, and the serum was collected and stored at −80 °C. 

### 2.5. Analysis of the Main Active Ingredients of Herbal Root Extract

**Figure 2:**
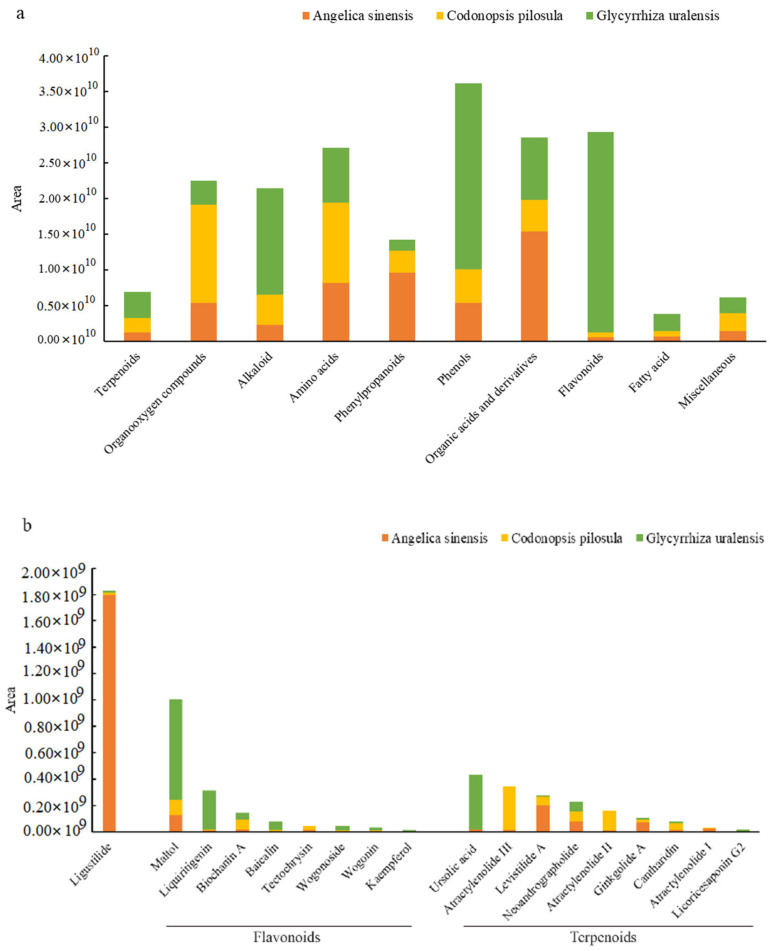

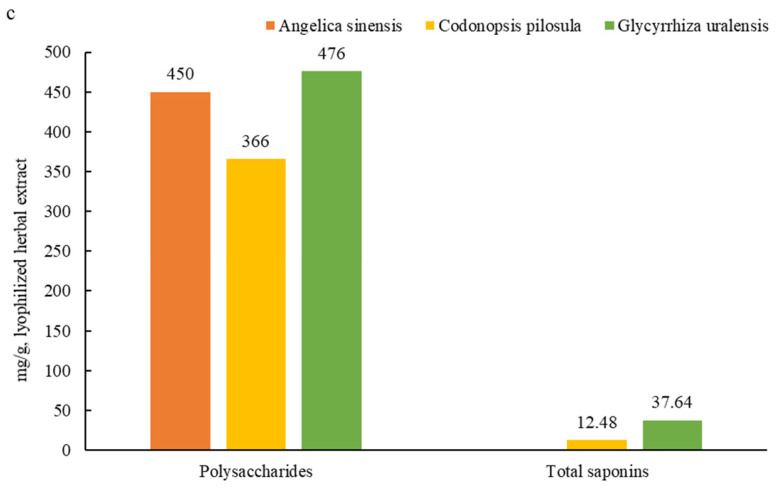
The main components of the three herbal extracts; (**a**), Main components in three herbal extracts detected by UHPLC-QE-MS; (**b**), ligustilide, flavonoids, and terpenoids of three herbal root extracts detected by UHPLC-QE-MS; (**c**), polysaccharides and total saponins (*Angelica sinensis* not measured) of three herbal root extracts.

Water extracts of the three herbal roots were condensed from 500 to 50 mL by a rotary evaporator (Lab Tech EV312, Beijing LabTech Instruments Co., Ltd., Beijing, China) and then lyophilized (Labconco FreeZone 7.5, Kingston, NY, USA). Polysaccharides were detected using the phenol-sulfuric acid method as described by Jiang et al. (2021), and the content was determined colorimetrically at 485 nm (Molecular Devices, SpectraMax M5, Thermo Fisher Scientific, Waltham, MA, USA), with glucose as a standard. Total saponins were extracted from *C. pilosula* and *G. uralensis* root extracts following Jiang et al. (2021), and saponin content was determined colorimetrically at 560 nm, with ginsenoside as a standard for *C. pilosula* and at 589 nm, with mono-ammonium glycyrrhizinate as a standard for *G. uralensis* [15]. The main active ingredients of the three herbal root extracts were identified in lyophilized herbal extract by ultra-high-performance liquid chromatography (UHPLC, Column: BEH0C18, 1.7 µm × 2.1 mm × 100 mm; Agilent Technologies 1290, Santa Clara, CA, USA) and mass spectrometry (MS, Type: Q Exactive Focus, Thermo Fisher Scientific Waltham, MA, USA). The MS conditions were described by Jiang et al. (2021). Peak detection, extraction, alignment, and integration of the mass data were processed by R package-XCMS. An in-house MS2 database (BiotreeDB) was applied for compound annotation. The relative contents of the compounds in the root extracts were determined from the peak area (Figure 2).

### 2.6. Analysis of Serum Biochemical Parameters

An automatic biochemical analyzer (BS-420, Mindray, Shenzhen, China) was used to measure serum concentrations of total protein (TP), albumin (ALB), urea nitrogen, triglycerides (TG), total cholesterol (TC), immunoglobulin A (IgA), immunoglobulin M (IgM), and immunoglobulin G (IgG) colorimetrically. ELISA kits (Beijing Sinoouk Institute of Biological Technology, Beijing, China) were used to determine the serum concentrations of free fatty acids (FFAs), insulin (INS), interleukin-2 (IL-2), interleukin-6 (IL-6), and tumor necrosis factor-α (TNF-α). ELISA kits (Nanjing Jiancheng Bioengineering Institute, Nanjing, China) were also used to measure serum concentrations of growth hormone (GH), superoxide dismutase (SOD), malondialdehyde (MDA), and glutathione peroxidase (GSH-Px).

### 2.7. Serum Metabolome Analysis, Bioinformatics, and Statistical Analysis

Serum samples collected on d 60 were used for metabolome analysis. The preparation of the sample and analysis followed Wang et al. (2021) [17]. 

One-way ANOVA (SPSS 25.0, SPSS Inc., Chicago, IL, USA) compared serum indices (including TP, ALB, BUN, IL-2, TNF-α, and MDA) among treatment groups, with the yak calf as the experimental unit. Where significance existed, the Tukey-adjusted *p* values separated means. Orthogonal polynomial contrasts determined whether responses to the herbal extracts were linear or quadratic with time when serial samples were collected. Significance was accepted at *p* < 0.05 and as a tendency for significance at 0.05 < *p* < 0.10.

We combined the results in positive and negative ion modes for the main active ingredients of herbal root extract detection. ProteoWizard converted the raw data to the mzXML format, which was processed with an in-house program that was developed using R and based on XCMS (https://xcmsonline.scripps.edu/landing_page.php?pgcontent=mainPage, accessed on 10 March 2021) for peak detection, extraction, alignment, and integration. An in-house MS2 database (BiotreeDB) was applied for metabolite annotation. The cut-off for annotation was set at 0.3.

Multivariate analysis of normalized data used SIMCA software (V16.0.2, Umea, Sweden). Principal component analysis (PCoA) was used to visualize trends in the samples and orthogonal partial least-squares discriminant analysis (OPLS-DA) examined differences between treatment groups and control groups to remove noise. Metabolites were plotted according to their importance in separating the two groups based on the OPLS-DA results, and each metabolite received a variable importance in the projection (VIP) value. Metabolites with VIP values exceeding 1.0 were taken as changed variables, and these were assessed using a Student’s *t*-test. A significantly differential metabolite between the groups was defined as a variable with a VIP > 1 and *p* < 0.05. Volcano plots were used to visualize the results. Kyoto Encyclopedia of Genes and Genomes (KEGG) metabolic pathways were mapped and analyzed by MetaboAnalyst 3.0 (http://www.metaboanalyst.ca/, accessed on 20 October 2021) based on the differentiated metabolites. Differential pathways were identified based on *p* < 0.05, and the cutoff of impact value from the topology analysis pathway was set to 0. 

## 3. Results

### 3.1. Main Active Ingredients in the Herbal Root Extracts

Of the three herbal root extract groups, *A. sinensis* had the highest contents of phenylpropanoids, organic acids, and their derivatives; *C. pilosula* had the highest contents of organo-oxygen and miscellaneous compounds; and *G. uralensis* had the highest contents of terpenoids, alkaloids, phenols, and flavonoids (Figure 2a). Ligustilide was the main flavonoid in *A. sinensis* (Figure 2b); biochanin A and tectochrysin were the main flavonoids in *C. pilosula*; and maltol, liquiritigenin, baicalin, and wogonoside were the main flavonoids in *G. uralensis*. Herbal extracts of *A. sinensis*, *C. pilosula*, and *G. uralensis* contained 450, 366, and 476 mg polysaccharides per g DM, respectively (Figure 2c), and *C. pilosula* and *G. uralensis* contained 12.5 and 37.6 mg total saponins per g DM, respectively. 

### 3.2. Effect of Herbal Extracts on Serum Biochemical Parameters

Serum TC concentration was lower in yaks consuming *A. sinensis* than in control yaks on d 30; serum TG concentration was lower in yaks consuming *G. uralensis* than control yaks on days 30 and 60; and serum TP concentration was higher in yaks consuming *A. sinensis* than in control yaks on d 15 (Table 1). There was no effect of dietary treatment on serum GH concentration, but there was an increasing trend (*p* = 0.065) of serum GH concentration with time in calves consuming *G. uralensis*. Calves consuming the three herbal root extracts had lower serum FFA concentrations on d 30 than control yaks, an effect that was maintained in calves consuming *C. pilosula* and *G. uralensis* on days 45 and 60. The serum ALB concentration of calves consuming *G. uralensis* was higher than control calves on days 15 and 60, and of calves consuming *A. sinensis* was higher (*p* < 0.05) than control calves on day 60. Serum BUN concentration increased quadratically in calves consuming *C. pilosula*, whereas, serum INS concentration decreased linearly with time in calves consuming *G. uralensis*. 

### 3.3. Effect of Herbal Extracts on Serum Immunity Indices

Generally, there was no effect of herbal extract intake on concentrations of serum immunity indices, except for serum TNF-α, which was greater (*p* < 0.05) in calves consuming *C. pilosula* and *G. uralensis* than in control calves, and in calves consuming *A. sinensis* than in control calves on d 45 (Table 2). Serum IgA concentration decreased with time in calves consuming *A. sinensis* (*p* < 0.05) and decreased quadratically (*p* = 0.001) in calves consuming *C. pilosula*. In general, there were linear or quadratic decreases (*p* < 0.05) in serum concentrations of IL-2 and IL-6 with time in all calf groups. Control calves consuming *A. sinensis* decreased (*p* < 0.05) serum TNF-α concentration quadratically with time. 

### 3.4. Effect of Herbal Extracts on Serum Antioxidant Capacity

Yak calves consuming root extract of *A. sinensis*, *C. pilosula*, and *G. uralensis* had lower (*p* < 0.05) serum MDA concentrations on d 15, consuming *C. pilosula* and *G. uralensis* had lower MDA concentrations on d 30, and consuming *G. uralensis* had lower MDA concentration on d 60 than control calves (Table 3). 

Serum GSH-Px concentrations were higher (*p* < 0.05) in calves consuming the three herbal root extracts on days 30, 45, and 60 than in control calves, with calves consuming *G. uralensis* having the highest concentration. Linear and quadratic increases in serum GSH-Px concentration with time (*p* < 0.05) emerged in control calves, and calves consuming *C. pilosula* and *G. uralensis*. Serum SOD concentration was not affected by diet (*p* = 0.40–0.80), but there were significant linear and quadratic increases in serum SOD concentration with time in all groups.

### 3.5. Identification and Quantification of LC-MS Compounds in Serum

**Figure 3:**
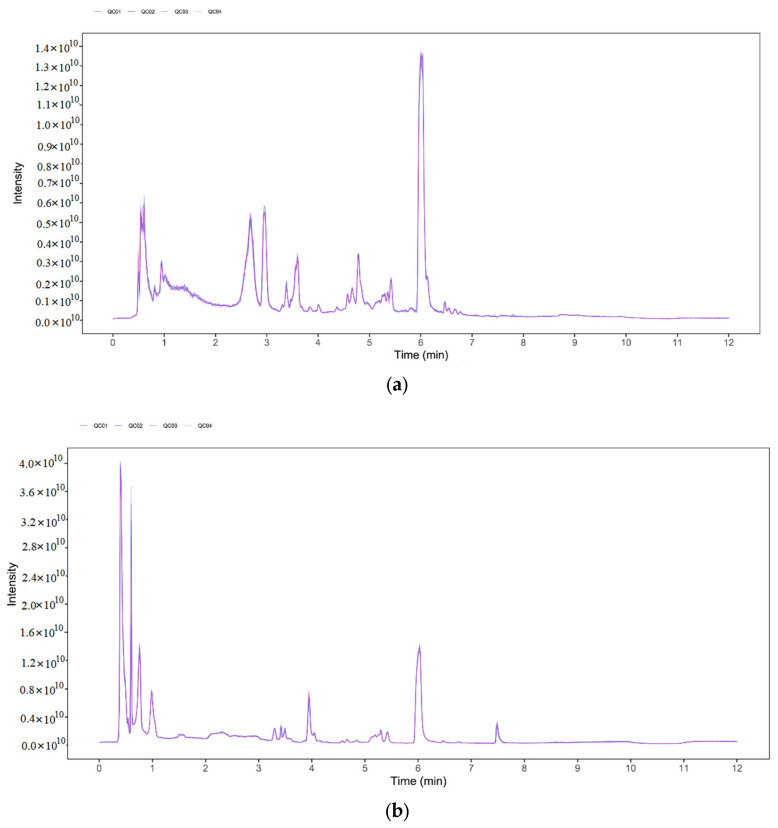
Total ion chromatograms (TICs) from all quality control (QC) samples detected by UHPLC-QE-MS in positive (**a**) and negative (**b**) modes. QC was prepared by mixing an equal aliquot of the supernatants from all samples. Q1 to Q4 were repeated samples to determine the stability of the instrument.

The stability and repeatability of the system were tested using four quality control (QC) samples. The ionization source of LC-QTOF/MS was electrospray ionization, including positive (POS) and negative (NEG) ion modes. There was substantial overlap in peak retention time and peak area of total ion chromatograms (TIC) from all QC samples, indicating that the analytical system was stable (Figure 3a,b). A total of 6293 valid peaks were identified in POS modes and 6748 in NEG modes for the serum. These peaks were matched for 301 (POS) and 234 (NEG) plasma metabolites based on an in-house MS2 database and the KEGG compound metabolomics library. The metabolites were integrated from the two modes, and a total of 511 qualitative metabolites were obtained. 

### 3.6. Metabolomic Profiles in Serum of Early Weaned Yak Calves

**Figure 4:**
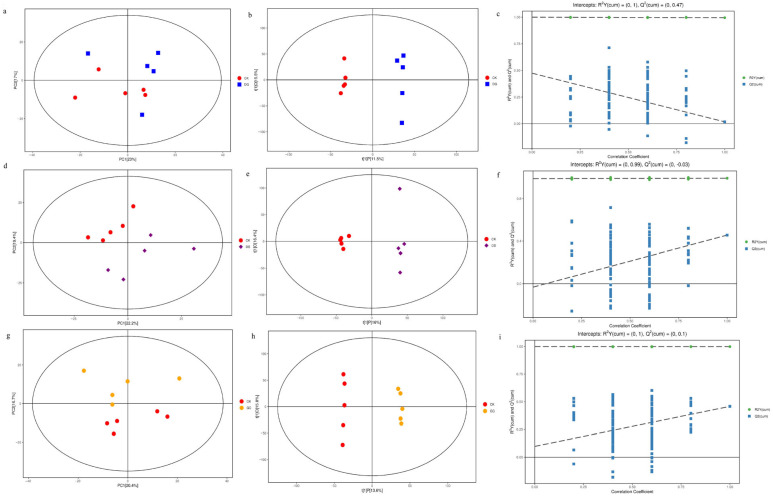
Principal component analysis (PCA) model score scatter plot, orthogonal partial least-squares discriminant analysis (OPLS-DA) model, and permutation test: (**a**–**c**) were derived from yak calves consuming *Angelica sinensis* and control calves, (**d**–**f**) were derived from yak calves consuming *Codonopsis* *pilosula* and control calves, and (**g**–**i**) were derived from yak calves consuming *Glycyrrhiza uralensis* and control calves.

Score plots of the PCA (Figure 4a) and OPLS-DA (Figure 4b) modes illustrated good separation between the control and *A sinensis* groups and all the samples fell within the 95% confidence interval (Hotelling’s T-squared ellipse). The permutation test was used for verification (Figure 4c). The Q^2^ was 0.00172, indicating that the predictive power of mode was not strong, which was in accord with the results that there were few significant plasma metabolites. For the *C. pilosula* and *G. uralensis* groups, the score plots of PCA (Figure 4d,g) and OPLS-DA (Figure 4e,h) modes demonstrated satisfactory modeling and predictive abilities between the control and treatment groups, and that all the samples fell within the 95% confidence interval (Hotelling’s T-squared ellipse). The permutation test was used for verification (Figure 4f,i), and the results indicated that the OPLS-DA model was suitable to test differences between the two groups, as there was no overfitting and good stability. Following the criteria of a *p* < 0.05 for the *t*-test and the VIP > 1 for the OPLS-DA model, significant differential metabolites between the control and treatment groups were screened from all identified metabolites. In the *A. sinensis* group, a total of 16 metabolites, mainly composed of amino acids, were identified and affirmed by combining MS/MS data and available biochemical databases. In the *C. pilosula* group, 29 metabolites, including amino acids, fatty acids, and glycerophospholipids, were identified. In the *G. uralensis* group, a total of 51 metabolites, including amino acids, glycerophospholipids, and fatty acids, were identified. 

**Figure 5:**
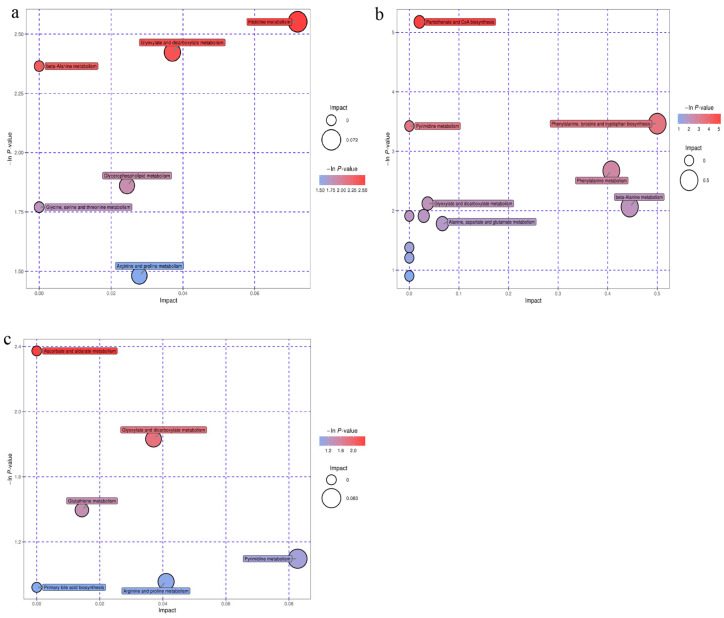
Pathway (each bubble represents a pathway) analysis of potential metabolite biomarkers. The larger the bubble, the larger the impact value; the darker the color of the bubble, the smaller the *p*-value: (**a**), *Angelica* *sinensis* group; (**b**), *Codonopsis pilosula* group; (**c**), *Glycyrrhiza uralensis* group.

There were 16 potential biomarkers in the *A. sinensis* group identified by the MetaboAnalyst website, with six major metabolic pathways, including histidine, glyoxylate and dicarboxylate, β-alanine, glycerophospholipid, glycine, serine and threonine, and arginine and proline metabolism. (Figure 5a). Twenty-nine potential biomarkers were identified in the *C. pilosula* group, with seven major pathways, including pantothenate and CoA biosynthesis, phenylalanine, tyrosine and tryptophan biosynthesis, pyrimidine metabolism, phenylalanine, β-alanine, glyoxylate and dicarboxylate, and alanine, asparate, and glutamate. (Figure 5b). Fifty potential biomarkers were identified in the *G. uralensis* group, with six major metabolic pathways, including glyoxylate and dicarboxylate, ascorbate and aldarate, glutathione, pyrimidine, arginine and proline metabolism, and primary bile acid biosynthesis (Figure 5c).

**Figure 6:**
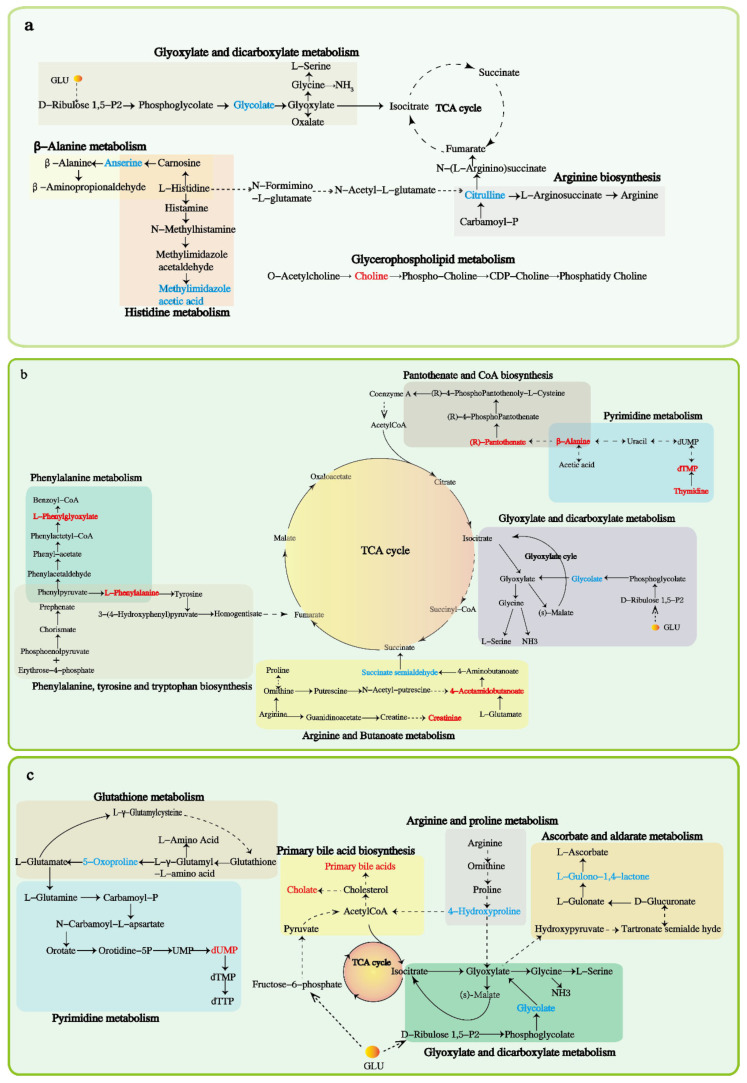
Network graph for the early weaned yak calves consuming herbal root extracts. The red metabolites represent down-regulation, and the blue metabolites represent up-regulation: (**a**), *Angelica* *sinensis* group; (**b**), *Codonopsis pilosula* group; (**c**), *Glycyrrhiza uralensis* group.

Compared to control calves, calves consuming root extract of *A. sinensis* down-regulated the biomarkers of choline in the metabolite pathways of glycine, serine, threonine, and glycerophospholipid; and up-regulated methylimidazole in histidine metabolism, glycolate in glyoxylate and dicarboxylate metabolism, citrulline in arginine biosynthesis, and anserine in β-alanine metabolism (Figure 6a). Root extract of *C. pilosula* down-regulated the biomarkers of l-phenylalanine in phenylalanine, tyrosine and tryptophan biosynthesis, phenylalanine in phenylalanine metabolism, pantothenate and β-alanine in pantothenate and CoA biosynthesis, 4-acetamidobutanoic acid in arginine and proline metabolism, and deoxythymidylic acid (dTMP) and thymidine in pyrimidine metabolism; and up-regulated succinic acid semialdehyde in butanoate metabolism, and glycolic acid in glyoxylate and dicarboxylate metabolism (Figure 6b). Root extract of *G. uralensis* down-regulated deoxyuridine monophosphate (dUMP) in pyrimidine metabolism and cholic acid and primary bile acids in primary bile acid biosynthesis; and up-regulated 5-oxoproline in glutathione metabolism, 4-hydroxyproline in arginine and proline metabolism, glycolic acid in glyoxylate and dicarboxylate metabolism, and L-gulonol-1,4-lactone in ascorbate and aldarate metabolism (Figure 6c). 

## 4. Discussion

### 4.1. Effect of Herbs on Serum Biochemical Indices

After one month of herbal intake, serum FFA concentrations were generally lower in the three yak calf groups consuming the herbal extracts than in control yaks. High serum FFA concentrations are related to fat mobilization and negative energy balance [18]. Consumption of *A. sinensis* polysaccharides decreased the serum concentration of total cholesterol in diabetic mice and accelerated serum FFA oxidation in mice [19,20]. In addition, botanical essential oils, a main component of *A. sinensis*, lowered the serum concentration of total cholesterol in pigs and sheep [21,22]. In the current study, serum TG concentrations were generally lower in yak calves consuming the herbal extracts, especially *G. uralensis*, than in control yak calves. Previous studies reported that administration of *G. uralensis* reduced hepatic TG levels in mice [23], while consuming saponins reduced serum TG concentrations in sheep and chickens [24,25]. Furthermore, ursolic acid, the main component of saponins (terpene) in *G. uralensis*, alleviated lipid accumulation by activating the AMPK signaling pathway [26]. The high level of total saponins in the root extract of *G. uralensis* could explain the particularly low serum TG concentration in yak calves consuming this herbal extract. Further studies are needed to examine the effect of the different active ingredients of the three herbs on fat storage and mobilization. 

The serum concentrations of total protein (TP) and albumin (ALB) reflect protein metabolism, mainly in the liver [27]. ALB is the most abundant serum protein, comprising 35 to 50% of the total, and is the major source of amino acids. It is important for the maintenance of homeostasis, contributing approximately 75% of the osmotic pressure of serum, for the transportation of substances, and for scavenging free radicals. The general increase in serum concentrations of TP and ALB by yak calves consuming herbal extracts could be related to polysaccharides. A previous study reported that polysaccharides of *Mesona chinensis* up-regulated the phosphorylation level of blood protein factors in mitogen-activated protein kinase signaling pathways [28]. 

Serum urea concentration did not differ among dietary treatments in the present study. The concentration of serum urea reflects protein and amino acid metabolism [29]. A decreased concentration of serum urea indicates increased nitrogen retention as a consequence of reduced protein catabolism in skeletal muscle [30]. The current results indicated that the three herbal water extracts did not affect body nitrogen metabolism. 

### 4.2. Effect of Herbs on Serum Immunity Indices

The consumption of *Astralagus* polysaccharides improved immunity indices in lambs [31,32]. The concentrations of the serum immunoglobulin IgA, IgG, and IgM were all numerically higher at all time periods in the yak calves consuming the three herbal extracts than in the control calves in the present study, albeit the differences were not statistically different. An increase in these immunoglobulins enhances cell-mediated or systemic humoral immunity [33]. Herbal extracts had no effect on serum IL-2 and IL-6 concentrations; however, both decreased linearly with time in the four groups. Low air temperature can cause a decrease in inflammatory cytokines, including IL-2 and IL-6 [34]. The present study took place from autumn to winter, that is, with decreasing air temperature (Figure 1), which is, most likely, the reason for the decrease in serum IL-2 and IL-6 concentrations with time in the yak calves. IL-2 is produced by activated T-cells and stimulates immune responses and the release of IL-1 and IL-6 [35]. Consuming herbal extracts had no effect on serum TNF-α concentration in the yak calves in the present study. Serum TNF-α plays an important role in protection against invading microorganisms and in the regulation of the inflammatory functions of macrophages [31,36]. The immune response is related to the duration and amount of herbal consumption [37]. Generally, the doses of the three herbal extracts consumed by the early-weaned yak calves in the current study did not affect their immune responses. 

### 4.3. Effect of Herbs on Serum Antioxidant Capacity

The serum concentration of MDA decreased in all groups consuming the herbal extracts when compared to control yak calves. Herbal compounds can prevent lipid peroxidation, and are used as synthetic antioxidants [38,39]. MDA is produced from the degradation of polyunsaturated fatty acids by lipid peroxidation, and is biologically active, possessing cytotoxic and genotoxic properties, which can be increased by reactive oxygen species (ROS) or other free radicals [40,41,42,43]. Consumption of an extract of dried *Hibiscus sabdarrif* flowers decreased MDA formation in rats, and flavonoids from natural plants inhibited MDA production [44,45], which could explain the reduction in serum MDA concentrations in yak calves consuming the three herbal root extracts in this study. 

The three herbal root extracts increased serum GSH-Px concentrations in the yak calves, which is consistent with the report that a water extract of *G. uralensis* fed to lambs increased GSH-Px gene activities in muscle tissue [46]. The serum concentration of SOD was numerically higher in the yak calves consuming extracts from the three herbs than in the control yak calves, albeit the differences did not differ statistically. Serum GSH-Px and SOD scavenge ROS and reduce oxidative stress [47]. Administration of polysaccharides from *A. sinensis* increased the GSH-Px level in shrimp [48], while polysaccharides from *C. pilosula* increased serum GSH-Px activity in fish [49]. Therefore, the polysaccharides in the herbal water extracts could explain the increase in serum GSH-Px and SOD concentrations [50,51].

### 4.4. Serum Metabolome Responses

A chronic lack of glycine could retard normal growth and have adverse effects on health and immunity in mammals [52]. Glyoxylate synthesizes glycine through transamination, primarily utilizing alanine by alanine-glyoxylate aminotransferase (AGT) [53], and glyoxylate can be converted into oxalate by lactate dehydrogenase [54]. The present study indicated that the root extract of *A. sinensis* could reduce the activity of AGT and enhance the activity of lactate dehydrogenase. Glyoxylate can be produced through glycolate oxidation by peroxisomal glycolate oxidase, and is ultimately converted to oxalate [53,55]). *C. pilosula* and *G. uralensis* inhibited the activity of glycolate oxidase, which may be the reason for the increased glycolate content in yak calves in the present study. Flavonoids (quercetin and kaempferol) display anti-nephrolithic properties by selective interaction with glycolate oxidase, mainly through the inhibition of peroxisomal glycolate oxidase by isolated flavonoids [55]. The flavonoid, tectochrysin, in *C. pilosula* and *G. uralensis* (low content in *A. sinensis*) most likely inhibited glycolate oxidase [55], but this premise requires further study. 

Ferulic acid was reported to increase antioxidant activity by increasing choline acetyltransferase and decreasing acetylcholinesterase activity [56], which is probably the reason for the down-regulating of choline in yak calves consuming *A. sinensis* in the present study. The up-regulation of citrulline in yak calves consuming *A. sinensis* is beneficial for the production of arginine. Nitrogen oxide is produced from arginine, and plays an important role in maintaining normal blood pressure and in scavenging pathogens in inflammatory diseases [57,58]. Anserine, the most abundant histidine-containing dipeptide in skeletal muscle in many vertebrates, reduces the risks of cardiovascular diseases [59,60]. The oxidation of N-methylhistamine by amine oxidase produces N-methylimidazole acetaldehyde, which is converted to methylimidazole acetic acid by aldehyde dehydrogenase [61]. Therefore, the components of *A. sinensis* regulate the two enzymes in the histidine metabolic pathway. Serum creatinine, a waste product from the breakdown of protein [62], is down-regulated with the consumption of *C. pilosula*. A previous study reported that polysaccharides from the stem of *C. pilosula* protected rats against renal injury, reduced blood creatinine levels, and improved renal glomerular function [63]. Active ingredients in the root extract of *C. pilosula* inhibit co-enzyme A synthesis, and affect the tricarboxylic acid cycle (TCA) cycle. The product of degraded thymidine, β-alanine, is metabolized into acetic acid [64,65], which can be diverted into pantothenic acid and co-enzyme A biosynthesis [66]. Pantothenate is a precursor of the fundamental enzyme co-factor co-enzyme A (CoA), which enters the TCA cycle [67]. The metabolome results indicated that consuming the root water extracts of *A. sinensis* and *C. pilosula* promoted energy and protein metabolism in yak calves, which is consistent with the findings of Ma et al. (2022) [68], who concluded that feeding *Astragalus membranaceus* root to pre-weaned dairy calves enhanced protein synthesis and gluconeogenesis. 

The root extract of *C. pilosula* down-regulated the production of phenylalanine in the present study. Phenylalanine, via phenylalanine hydroxylase, produces tyrosine, which produces the neurotransmitter dopamine [69]. The ethanol eluent of *Semen ziziphi* and *Radix polygalae* down-regulated phenylalanine, mainly due to the sedative-hypnotic role of the flavonoid and saponin contents [70]. Glycosides of *C. pilosula* act as a sedative agent [71]. Thymidine, along with arachidonic acid and 2′-deoxycytidine, was identified as an indicator of cardiotoxicity, as an increase in thymidine was related to cardiotoxic drugs [72]. Thymidine can be converted into dUMP and dTMP by the catalysis of enzymes in pyrimidine metabolism. The current study down-regulated pyrimidine metabolism in yak calves consuming *C. pilosula* and *G. uralensis* root extract, which may be related to the protection of the heart and kidney.

In the current study, the up-regulating of 5-oxoproline, a product of glutathione metabolism, indicates oxidative stress, and is generally accompanied by the elevation of ascorbate [73,74], a typical antioxidant [75]. L-gulono-1,4-lactone is the intermediate in the synthesis of L-ascorbate by L-gulono-1,4-lactone oxidase [76]. Therefore, the root extract of *G. uralensis* enhances the antioxidant capacity of yak calves by promoting ascorbate production. It was reported that 4-hydroxyproline plays a key role in collagen stability, and in supporting muscle growth in animals [77,78]. The root extract of *G. uralensis* could potentially be beneficial for the growth rate of yak calves. *G. uralensis* root extract possesses detoxification properties by its regulatory effect on the plasma bile acid levels of mice, which up-regulates free and glycine bile acids and down-regulates taurine bile acids [79]. In the current study, yak calves consuming *G. uralensis* root extract down-regulated primary bile acids and cholic acid. 

## 5. Conclusions

It was concluded that the root extracts of *A. sinensis*, *C. pilosula*, and *G. uralensis* consumed as dietary supplements by early-weaned yak calves enhanced antioxidant capacity, and increased energy and nitrogen metabolism. However, the immune-stimulating effect was not significant with the doses of the three herbal water extracts consumed by the yak calves in the current study. The three herbal root extracts up-regulated the metabolite, glycolate; *A. sinensis* down-regulated the metabolism of β-alanine, arginine, and histidine; and *C. pilosula* and *G. uralensis* had an inhibiting effect on pyrimidine metabolism. Further studies are needed to determine the optimal level of herb extract to be offered to the yak calves.

## Figures and Tables

**Table 1:** Serum biochemical parameters of early weaned yak calves consuming different herbal supplements.

Items ^ǂ^	Day	Treatment				SEM	*p*
		Control	*Angelica sinensis*	*Codonopsis pilosula*	*Glycyrrhiza uralensis*		
TC, mmol/L	15	1.13	1.18	1.18	1.15	0.100	0.999
	30	1.52 ^a^	0.85 ^b^	1.10 ^ab^	1.12 ^ab^	0.075	0.001
	45	1.47	1.39	1.15	1.30	0.072	0.460
	60	1.31	1.23	1.15	0.98	0.091	0.616
*p* ^#^	L	0.539	0.395	0.959	0.602		
	Q	0.287	0.697	0.969	0.599		
TG, mmol/L	15	0.26	0.26	0.27	0.19	0.018	0.317
	30	0.29 ^a^	0.28 ^ab^	0.26 ^ab^	0.22 ^b^	0.014	0.017
	45	0.35	0.30	0.29	0.28	0.023	0.566
	60	0.32 ^a^	0.24 ^ab^	0.26 ^ab^	0.17 ^b^	0.021	0.048
*p*	L	0.196	0.840	0.998	0.862		
	Q	0.266	0.103	0.974	0.052		
TP, g/L	15	47.1 ^b^	63.0 ^a^	58.2 ^ab^	56.0 ^ab^	1.987	0.017
	30	56.8	60.9	54.0	59.9	2.058	0.708
	45	58.3	60.9	60.3	63.3	2.298	0.920
	60	49.2	60.9	54.5	53.1	1.815	0.601
*p*	L	0.581	0.314	0.756	0.756		
	Q	0.043	0.612	0.942	0.321		
ALB, g/L	15	27.5 ^b^	31.7 ^ab^	32.7 ^ab^	33.6 ^a^	0.846	0.017
	30	24.7	27.4	27.6	29.7	0.565	0.114
	45	24.6	28.5	27.4	29.9	1.092	0.115
	60	25.0 ^b^	31.9 ^a^	28.4 ^ab^	30.3 ^a^	1.104	0.041
*p*	L	0.545	0.502	0.508	0.630		
	Q	0.138	0.085	0.027	0.204		
GH, ng/mL	15	5.31	4.89	6.05	5.29	0.205	0.187
	30	4.56	5.36	4.77	4.72	0.181	0.482
	45	5.77	6.74	7.18	7.25	0.417	0.683
	60	5.03	6.06	6.50	6.86	0.298	0.130
*p*	L	0.759	0.151	0.176	0.065		
	Q	0.911	0.289	0.393	0.198		
FFA, mmol/L	15	0.38	0.39	0.33	0.36	0.116	0.321
	30	0.43 ^a^	0.36 ^b^	0.37 ^b^	0.37 ^b^	0.011	0.021
	45	0.46 ^a^	0.44 ^ab^	0.39 ^b^	0.37 ^b^	0.015	0.018
	60	0.48 ^a^	0.37 ^ab^	0.39 ^ab^	0.34 ^b^	0.019	0.021
*p*	L	0.004	0.858	0.117	0.751		
	Q	0.014	0.728	0.047	0.074		
INS, μIU/mL	15	12.06	13.21	12.77	13.60	0.291	0.282
	30	9.36	10.14	11.89	11.20	0.499	0.182
	45	12.42	13.14	13.73	13.13	0.358	0.724
	60	9.23	10.10	9.81	9.58	0.160	0.320
*p*	L	0.243	0.255	0.257	0.024		
	Q	0.501	0.491	0.132	0.063		
BUN, mmol/L	15	3.11	3.21	2.70	3.36	0.160	0.605
	30	3.25	3.11	3.03	3.33	0.116	0.708
	45	3.43	3.22	3.35	3.33	0.105	0.081
	60	3.71	3.76	3.25	3.52	0.143	0.643
*p*	L	0.094	0.098	0.079	0.704		
	Q	0.199	0.166	0.008	0.860		

^ǂ^ GLU, glucose; TC, total cholesterol; TG, triglyceride; TP, total protein; GH, growth hormone; FFA, free fatty acid; ALB, albumin; BUN, blood urea nitrogen; INS, insulin. ^a^^,^^b^ means within a row with different superscripts differ from each other (*p* < 0.05). ^#^ L, linear effect of day; Q, quadratic effect of day.

**Table 2:** Serum immunity indices of early weaned yak calves consuming different herbal extract supplements.

Items ^ǂ^	Day	Treatment				SEM	*p*
		Control	*Angelica sinensis*	*Codonopsis pilosula*	*Glycyrrhiza uralensis*		
IgA, g/L	15	0.80	0.86	0.85	0.85	0.009	0.862
	30	0.72	0.78	0.76	0.81	0.016	0.293
	45	0.73	0.79	0.75	0.79	0.014	0.273
	60	0.75	0.76	0.84	0.81	0.016	0.195
*p* ^#^	L	0.134	0.002	0.582	0.152		
	Q	0.059	0.004	0.001	0.121		
IgG, g/L	15	10.6	11.7	11.8	11.8	0.328	0.486
	30	10.6	10.9	11.1	11.5	0.274	0.634
	45	11.2	11.8	12.6	11.7	0.204	0.107
	60	11.4	12.1	10.7	10.3	0.369	0.084
*p*	L	0.278	0.450	0.670	0.097		
	Q	0.560	0.539	0.595	0.159		
IgM, g/L	15	2.59	3.46	2.94	3.37	0.163	0.253
	30	2.72	2.76	3.09	3.02	0.056	0.292
	45	2.87	2.96	3.16	2.81	0.088	0.611
	60	2.75	2.85	2.76	2.90	0.065	0.763
*p*	L	0.502	0.13	0.491	0.107		
	Q	0.679	0.11	0.252	0.148		
IL-2, pg/mL	15	332	334	329	324	2.72	0.757
	30	253	232	246	240	4.73	0.403
	45	271	279	267	285	4.85	0.173
	60	188	185	200	190	2.00	0.657
*p*	L	<0.001	0.001	<0.001	0.001		
	Q	<0.001	0.007	0.003	0.003		
IL-6, pg/mL	15	137.0	141.4	143.2	136.6	1.60	0.483
	30	118.3	110.5	119.6	118.2	2.83	0.746
	45	128.6	129.6	127.5	126.6	1.16	0.447
	60	100.2	95.8	91.6	93.19	2.368	0.401
*p*	L	<0.001	0.007	0.009	0.004		
	Q	<0.001	0.031	0.039	0.016		
TNF-α, pg/mL	15	64.4	64.6	66.1	62.1	0.913	0.214
	30	52.8	51.6	56.8	54.9	0.964	0.251
	45	56.8 ^b^	54.6 ^b^	64.2 ^a^	64.0 ^a^	1.556	0.016
	60	53.5	58.0	56.8	58.6	2.278	0.120
*p*	L	0.889	0.825	0.131	0.523		
	Q	0.001	0.004	0.319	0.259		

^ǂ^ IgA, immunoglobulin A; IgG, immunoglobulin G; IgM, immunoglobulin M; IL-2, interleukin-2; IL-6, interleukin-6; TNF-α, tumor necrosis factor. ^a,b^ means within a row with different superscripts differ from each other (*p* < 0.05). ^#^ L, linear effect of day; Q, quadratic effect of day.

**Table 3:** Serum antioxidant capacities of early weaned yak calves consuming different herbal extract supplements.

Items ^ǂ^	Day	Treatment				SEM	*p*
		Control	*Angelica sinensis*	*Codonopsis pilosula*	*Glycyrrhiza uralensis*		
MDA, nmol/mL	15	2.90 ^a^	1.94 ^b^	1.98 ^b^	2.25 ^b^	0.129	0.003
	30	3.08 ^a^	2.75 ^ab^	2.10 ^c^	2.38 ^bc^	0.122	0.002
	45	2.78	2.23	2.35	2.38	0.108	0.336
	60	1.91 ^a^	1.55 ^ab^	1.68 ^ab^	1.35 ^b^	0.069	0.004
*p* ^#^	L	0.216	0.002	0.216	0.022		
	Q	0.003	0.001	0.003	0.001		
GSH-Px, U/mL	15	397	445	436	428	9.2	0.299
	30	310 ^c^	406 ^b^	448 ^ab^	466 ^a^	18.9	<0.001
	45	342 ^c^	428 ^b^	411 ^b^	470 ^a^	14.4	<0.001
	60	324 ^c^	398 ^b^	383 ^b^	553 ^a^	15.6	<0.001
*p*	L	0.051	0.112	0.007	<0.001		
	Q	0.026	0.292	0.009	<0.001		
SOD, U/mL	15	85.2	85.1	88.2	87.3	0.78	0.422
	30	91.2	92.2	92.6	93.6	0.85	0.841
	45	91.9	93.1	94.7	94.7	0.69	0.443
	60	92.4	92.8	94.5	92.8	0.59	0.687
*p*	L	0.018	0.022	0.005	0.024		
	Q	0.023	0.007	0.008	0.002		

^ǂ^ MDA, Malondialdehyde; GSH-Px, Glutathione peroxidase; SOD, Superoxide dismutase. ^a–c^ means within a row with different superscripts differ from each other (*p* < 0.05). ^#^ L, linear effect of day; Q, quadratic effect of day.

## Data Availability

Not applicable.
